# Multifunctional Zn-Doped ITO Sol–Gel Films Deposited on Different Substrates: Application as CO_2_-Sensing Material

**DOI:** 10.3390/nano12183244

**Published:** 2022-09-19

**Authors:** Mariuca Gartner, Mihai Anastasescu, Jose Maria Calderon-Moreno, Madalina Nicolescu, Hermine Stroescu, Cristian Hornoiu, Silviu Preda, Luminita Predoana, Daiana Mitrea, Maria Covei, Valentin-Adrian Maraloiu, Valentin Serban Teodorescu, Carmen Moldovan, Peter Petrik, Maria Zaharescu

**Affiliations:** 1 Institute of Physical Chemistry “Ilie Murgulescu”, Romanian Academy, 202 Splaiul Independentei, 060021 Bucharest, Romania; 2 Renewable Energy Systems and Recycling, Transilvania University of Brasov, Eroilor 29, 500036 Brasov, Romania; 3 National Institute of Materials Physics, 405 bis Atomistilor Street, 077125 Magurele-Ilfov, Romania; 4 National Institute for Research and Development in Microtechnologies, 077190 Bucharest, Romania; 5 Centre for Energy Research, Hungarian Academy of Sciences, Konkoly-Thege Str. 29-33, H-1121 Budapest, Hungary; 6 Department of Electrical and Electronic Engineering, Institute of Physics, Faculty of Science and Technology, University of Debrecen, H-4032 Debrecen, Hungary

**Keywords:** sol–gel films, Zn-doped ITO thin films, optical properties, electrical properties, microstructure, gas testing

## Abstract

Undoped and Zn-doped ITO (ITO:Zn) multifunctional thin films were successfully synthesized using the sol–gel and dipping method on three different types of substrates (glass, SiO_2_/glass, and Si). The effect of Zn doping on the optoelectronic, microstructural, and gas-sensing properties of the films was investigated using X-ray diffraction (XRD), atomic force microscopy (AFM), scanning electron microscopy (SEM), transmission electron microscopy (TEM), spectroscopic ellipsometry (SE), Raman spectroscopy, Hall effect measurements (HE), and gas testing. The results showed that the optical constants, the transmission, and the carrier numbers were correlated with the substrate type and with the microstructure and the thickness of the films. The Raman study showed the formation of ITO films and the incorporation of Zn in the doped film (ITO:Zn), which was confirmed by EDX analysis. The potential use of the multifunctional sol–gel ITO and ITO:Zn thin films was proven for TCO applications or gas-sensing experiments toward CO_2_. The Nyquist plots and equivalent circuit for fitting the experimental data were provided. The best electrical response of the sensor in CO_2_ atmosphere was found at 150 °C, with activation energy of around 0.31 eV.

## 1. Introduction

Indium tin oxide (ITO) materials can be manufactured to exhibit high transmittance and good electrical properties (high carrier concentration and mobility), which make them attractive for different applications. ITO thin films prepared using various (physical and chemical) methods are used in a large range of applications such as solar cells [1,2,3], transparent electrodes in plasma displays panels [4], electroluminescent devices or light-emitting diodes (OLEDs) [5,6,7], transparent conducting oxides (TCO) [8,9], and gas sensors [10]. The targeted gases are especially hazardous ones, such as toluene [11,12], hydrogen [13,14,15,16], ammonia [17,18,19,20,21], chlorine [22,23], NO_2_ [24,25,26], CO_2_ [15,24,25,27], and CO [28]. The sensors can also detect humidity [29,30]. For these applications, the films were optimized by controlling various parameters such as the annealing temperature [31,32,33,34], substrate type [35,36,37,38], deposition method [39,40,41,42,43], and doping [44,45,46,47,48]. Regarding the deposition method, the ITO thin films were most frequently prepared using physical methods. However, over time, researchers have studied and approached ITO thin-film preparation using chemical methods such as sol–gel [49,50] and low-temperature combustion synthesis [51].

The Zn-doped ITO (ITO:Zn) films reported so far in the literature were deposited using physical methods, especially magnetron sputtering on glass substrate [52], quartz [53], and plastic foils [44,54]. A more recent paper presented the thermoelectric properties of Zn-doped ITO thin films with various Zn concentrations prepared using the magnetron co-sputtering method [55]. Accordingly, there are very few published papers concerning Zn-doped ITO thin films prepared using sol–gel methods [56,57].

To this end, on the basis of our experience [58,59,60,61,62] in this field, the current paper presents the results on ITO:Zn films prepared using the sol–gel method, a highly economical and up-scalable chemical method. This is due to the uniform distribution of the dopant in the matrix, as well as the overall uniformity of the films, regardless of the deposition area. In the present study, we focused on the effects of 4% Zn doping on the optoelectronic properties of ITO films. A comparative analysis of undoped and Zn-doped films was carried out for the films deposited using the sol–gel and dipping methods on glass, SiO_2_/glass, and Si substrates. Comprehensive analyses regarding the structural, morphological, optical, and electrical properties of these films were performed using X-ray diffraction (XRD), atomic force microscopy (AFM), scanning electron microscopy (SEM), transmission electron microscopy (TEM), spectroscopic ellipsometry (SE), Raman spectroscopy, and Hall effect (HE) measurements. The multifunctional characteristic of the prepared films was evidenced by examining the TCO characteristics and by conducting gas-sensing tests for CO_2_ (at the threshold indoor concentration of 1000 ppm in air).

## 2. Materials and Methods

### 2.1. Film Preparation

**Figure 1:**
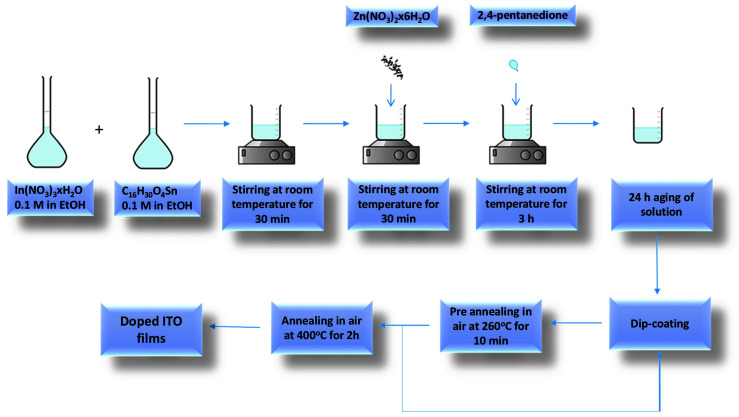
Flowchart for the preparation of the ITO:Zn thin films.

The Zn-doped ITO films (further denoted as ITO:Zn) were prepared using the sol–gel method with solutions of 0.1 M concentration. The following reagents were selected: indium(III) nitrate as In_2_O_3_ source, tin(II) 2-ethylhexanoate as SnO_2_ source, zinc nitrate as dopant source, 2,4-pentanedione as chelating agent, and ethanol as solvent. The flowchart for ITO thin-film preparation is presented in the Figure 1; the reaction took place under stirring at room temperature for 3 h. 

Before deposition, the solutions were aged for 24 h in air. Final ITO films were obtained through five successive depositions using the dip-coating method on microscopic glass, SiO_2_/glass, and Si substrates with a withdrawal rate of 5 cm/min. After each deposition, a consolidation treatment of the films was performed at 260 °C for 10 min. After the last deposition, the films were thermally heated at 400 °C for 2 h, using a heating rate of 5 °C/min. In the case of SiO_2_-covered glass substrate (SiO_2_/glass), the SiO_2_ protective film was prepared using the sol–gel method as described in our previous paper [58].

### 2.2. Film Characterization

The microstructure and surface composition of the films were studied using SEM with a field-emission gun microscope FEI Quanta 3D (Hillsboro, OR, USA) equipped with an energy-dispersive X-ray (EDX) spectrometer. Secondary electron micrographs were recorded in high-vacuum mode using an Everhart–Thornley detector, at accelerating voltages between 5 and 10 kV. The standardless ZAF corrected method was used for quantitative analysis of EDX spectra.

Structural characterization using TEM was performed with a JEOL ARM200F analytical electron microscope (Tokyo, Japan) operated at 200 kV and equipped with a JEOL JED-2300T unit to acquire EDX spectra or maps for elemental investigation. The sample preparation was performed using the classical cross-section method by cutting 2 × 1 mm^2^ pieces, gluing them face to face, mechanical polishing, and final ionic thinning using a Gatan PIPS System. EDX mapping in scanning TEM (STEM) mode was also performed to map the distribution of the component elements in the ITO films.

The crystallinity of the thermally treated ITO:Zn films was studied using XRD. The measurements were carried out using Rigaku Ultima IV equipment, with Cu K_α_ radiation and a fixed power source (40 kV and 30 mA). The diffractometer was set in the condition of grazing incidence X-ray diffraction (GIXD) with ω = 0.3°. The films were scanned at a rate of 5°/min over a range of 2θ = 20°–90°. Crystallite size was determined using Scherrer’s formula.

AFM measurements were conducted to examine the surface morphology. The measurements were carried out in noncontact mode, with XE-100 apparatus from Park Systems, using sharp tips. The topographical 2D AFM images were taken over an area of 1 × 1 µm^2^. The images were processed with XEI (v.1.8.0) Image Processing Program developed by Park Systems regarding the tilt correction and the evaluation of the root-mean-square roughness.

SE measurements were performed to obtain the thickness, optical constants, and optical bandgap (E_g_) in the UV/Vis/NIR spectral range using a J. A. Woollam Co. Inc. variable-angle spectroscopic ellipsometer. Measurements were performed at room temperature, using an incidence angle of 70°, in a 300–1700 nm spectral range, with a 10 nm wavelength step. The WASE program from Woollam was used for multiparameter fitting in which an iterative least-squares method was used for minimizing the difference (mean square error—MSE) between the experimental and the theoretical data. The film thickness and the refractive index (n) were obtained from the ellipsometric data analysis with an accuracy of ±0.2 nm and ±0.005, respectively. The optical transmission measurements were performed at 0° incidence angle on the same apparatus.

Raman spectra were measured at room temperature using a LabRAM equipment (Horiba Jobin Yvon, Tokyo, Japan), with the UV/Raman line (λ_exc_ = 325 nm) of a He/Cd laser to excite the Raman spectra; the laser spot size was around 1–2 μm. Measurements were performed under the microscope using an NUV 40× objective, covering the Raman shift range between 300 and 800 cm^−1^.

HE measurements were carried out on an HMS-5000 instrument from Ecopia (Chandler Heights, Arizona, AZ, USA) with an applied magnetic field of 0.55 T at room temperature, in Van der Pauw configuration. The Hall parameters such as charge carrier concentration, mobility, and resistivity were obtained, and their evolution was correlated with the Zn doping of the ITO thin films.

Gas sensing experiments of the ITO:Zn films deposited on Si were investigated using the four-point probe method on a Probostat standard cell for impedance measurements. The samples were placed in a controlled atmosphere with a continuous gas flow of 177 mL/min (provided by a calibrated system of mass-flow controllers—MFCs) [63]. The gases (synthetic air and CO_2_) were mixed inside a special vessel placed along the gas flow line before the inlet of the impedance measurement cell. The resistance of the films was influenced by the composition of the gaseous atmosphere inside the experimental cell. The resistance variations were recorded using a Solartron SI 1260 impedance/gain phase analyzer, by applying an AC voltage of 500 mV at different frequencies (5 MHz–100 Hz) on the sample. The testing gas was CO_2_, at 1000 ppm in air, and the so-called “working temperature” was varied between 100 and 300 °C. The motivation for choosing CO_2_ as a test gas lies in the fact that it represents 77% of the greenhouse gas emissions, although it is a colorless, odorless, and harmless gas within a certain concentration range. According to European standards, the maximum allowed level of CO_2_ in the indoor air of buildings is between 350 and 2500 ppm, but it is recommended to avoid exceeding the threshold of 1000 ppm [64].

## 3. Results and Discussion

### 3.1. Scanning Electron Microscopy (SEM)

**Figure 2:**
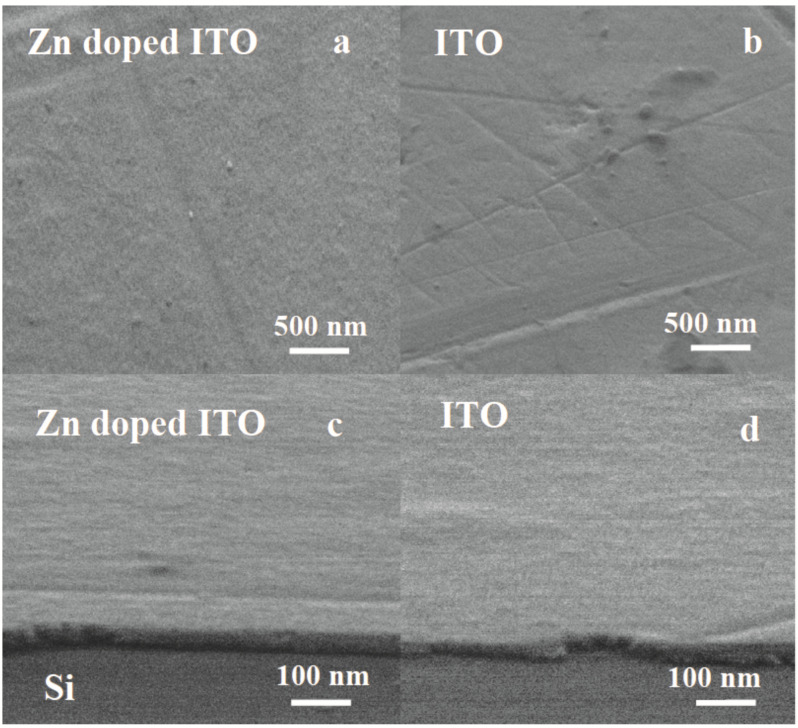
SEM micrographs at different magnification: (**a**,**b**) 100,000×, surface top view; (**c**,**d**) 500,000×, edge view. (**a**–**c**) ITO:Zn and (**b**–**d**) undoped ITO films. The darker gray area below the film edge in (**c**,**d**) is the underlying Si substrate.

SEM micrographs of the ITO:Zn films after five successive depositions on Si substrate are presented in Figure 2a (surface top view) and Figure 2c (edge view), while, for the undoped film, the corresponding SEM micrographs are shown in Figure 2b (top view) and Figure 2d (edge view).

The undoped and Zn-doped ITO films were very similar regarding their microstructure and thickness. They were continuous and presented low surface roughness. The thickness of the films measured from the edge view micrographs was approximately 30 nm for both ITO:Zn and undoped ITO films.

### 3.2. Chemical Characterization (EDX)

**Figure 3:**
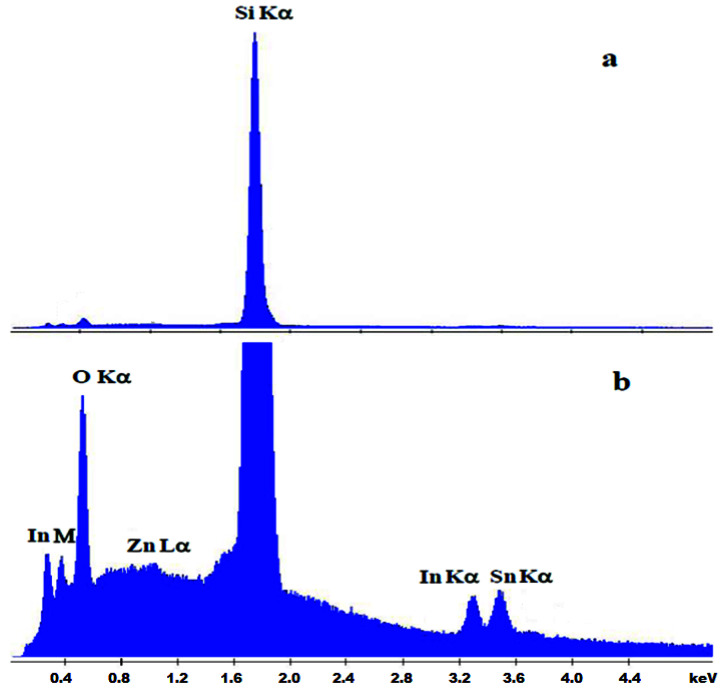
EDX spectrum of ITO:Zn film on Si: (**a**) full spectrum, showing the Si K_α_ line from the substrate and small contributions of the O K_α_, In M, In K_α_, Sn K_α_, and Zn L_α_ peaks from the film; (**b**) magnified view of the peaks from the film.

The chemical composition of doped and undoped ITO samples was analyzed using EDX. A low SEM accelerating voltage of 10 kV, sufficient to excite the L_α_ bands of In and Sn, was used to minimize the signal from the substrate. We estimate that, even at this low voltage, the depth of penetration inside the material in the analyzed area, according to Castaing’s expression, exceeded one micron, and the signal from the substrate was one or two orders of magnitude stronger than that from the film. Because the Zn L_α_ band from the doped film overlapped with the Na K_α_ band from the sodium-silicate glass substrate, it was difficult to assess the amount of Zn incorporated into the doped samples deposited on the glass or on the SiO_2_/glass substrates. Therefore, the results are presented for the doped and undoped films deposited on the Si substrate (Table 1). Figure 3 shows the typical EDX spectrum of a Zn-doped film on Si substrate. Figure 3a illustrates how the Si signal from the substrate dominates the spectrum, confirming the small film thickness, while Figure 3b shows an enlarged view that magnifies the features of the film, confirming the presence of In, Sn, O, and Zn. The first unmarked peak corresponds to C K_α_ from surface carbon. Table 1 shows the quantification of the elements In, Sn, and Zn from the EDX spectrum of the films deposited on Si. For quantification, we did not consider the contribution of the peaks from Si, O, and C. Zn was incorporated into the doped film, in a Zn/(In + Sn) ratio of 4/96. It was not possible to determine whether the incorporation of Zn took place by substituting In or Sn into the lattice or as an interstitial ion.

### 3.3. Transmission Electron Microscopy (TEM) and Energy-Dispersive X-ray (EDX)

**Figure 4:**
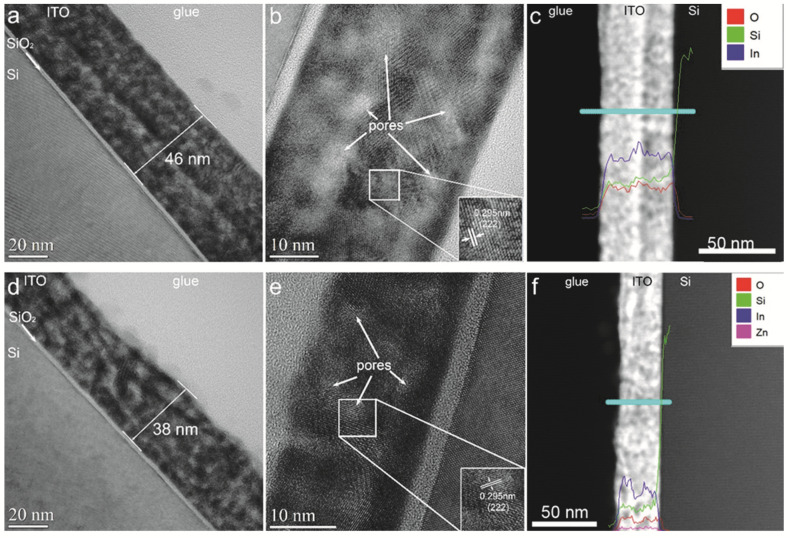
CTEM images (**a**,**d**), HRTEM images (**b**,**e**), and EDX line scan in HAADF image (**c**,**f**) for ITO and ITO:Zn films, respectively.

Conventional TEM (CTEM) images show that the ITO film had a thickness of 46 nm (Figure 4a), while the ITO:Zn film had a thickness of 38 nm (Figure 4d). In the insets of high-resolution TEM images (Figure 4b,e), lattice fringes of 0.295 nm corresponding to (222) planes of ITO are clearly visible. 

Moreover, in CTEM and HRTEM images, the contrast in the ITO films varied with brighter and darker regions. This suggests the existence of pores (indicated with white arrows in HRTEM images). To gain more information about the Zn distribution in the doped ITO film, we performed an EDX line profile in STEM mode on the undoped and doped ITO films. Using this method, both spatial information and spectral information were acquired simultaneously in each pixel. A convergent beam with a diameter of approximately 0.3 nm was used to scan 50 points forming the line for 30 s each. The X-ray signal from each point of the scan was collected by the detector. In the end, the profile of the atomic concentration for each of the chemical elements of interest (O, Si, In, and Zn) is displayed. In the high-angle annular dark field (HAADF) image for undoped ITO film (Figure 4c), a region with brighter contrast in the middle can be observed. According to the EDX profiles of the elements, it can be noted that, in this region, there was a higher concentration of In. EDX profiles for doped ITO film (Figure 4f) demonstrate that the Zn distribution along the film was uniform, and that the regions with darker contrast in HAADF image corresponded to the pores in CTEM images because the atomic concentration of In in these regions decreased.

### 3.4. Structural Characterization (XRD)

**Figure 5:**
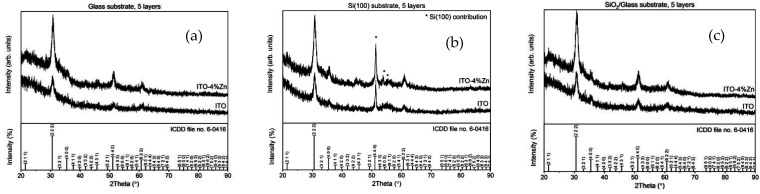
XRD patterns of ITO films undoped and doped with 4% Zn deposited on (**a**) glass, (**b**) Si, and (**c**) SiO_2_/glass.

Figure 5a–c show the XRD patterns of ITO and ITO:Zn films deposited on the three different substrates (glass, Si, and SiO_2_/glass). All thin films were polycrystalline, and the diffraction lines could be indexed to a cubic bixbyite-type In_2_O_3_ structure (ICDD file no. 6-0416). There were no diffraction lines corresponding to the other phases, indicating that the Zn dopant was completely incorporated into the Sn-doped In_2_O_3_ lattice. Regardless of the substrate, the ITO:Zn thin films possessed an increased degree of crystallinity. For the films deposited on Si, a contribution from the silicon substrate can be noted (marked by an asterisk in the Figure 5b), overlapping the (440) plane of the bixbyite-type structure.

The dopant also influenced the interplanar spacing. A shift to higher 2θ angles can be noted in Table 2, corresponding to smaller d-values for the doped thin film when the same substrate was used. 

This decrease in interplanar spacing can be attributed to the substitution of more In^3+^ by Zn^2+^, in addition to Sn^4+^ replacing In^3+^. The cation radius of Zn^2+^ (0.74 Å) is smaller than that of In^3+^ (0.8 Å) in their octahedral coordination. A decrease in the structural parameters for both co-dopants (Zn and Sn) was also noted [65]. The replacement of In^3+^ by Zn^2+^ marks the successful doping of the ITO samples with Zn, which can be expected to positively impact the electrical properties of the films (as further confirmed by HE measurements).

In terms of crystallite sizes, the broadening of the (222) diffraction line of the thin films deposited on glass was noticeably wider than that of the other two samples, deposited on SiO_2_/glass and Si (100). Calculated using Scherrer’s formula (Equation (1)) from the full width at half maximum (FWHM), for the (222) crystal plane only, the crystallite size for the sample deposited on glass was smaller, for both the undoped and the Zn-doped samples (Table 2). For the same type of substrate, the crystallite size of the doped samples was slightly smaller. This behavior could be explained by the presence of the Zn^2+^ dopant, which might have generated a higher amount of nucleation centers and, therefore, a reduction in the crystallite size of the doped thin films.

(1) $$D=\frac{0.94\times \lambda }{\beta \times cos\theta },$$

where *D* is the mean size of the ordered crystalline domains, λ is the X-ray wavelength, β is the line broadening at half the maximum intensity (FWHM), and θ is the Bragg angle.

### 3.5. Morphological Characterization (AFM)

**Figure 6:**
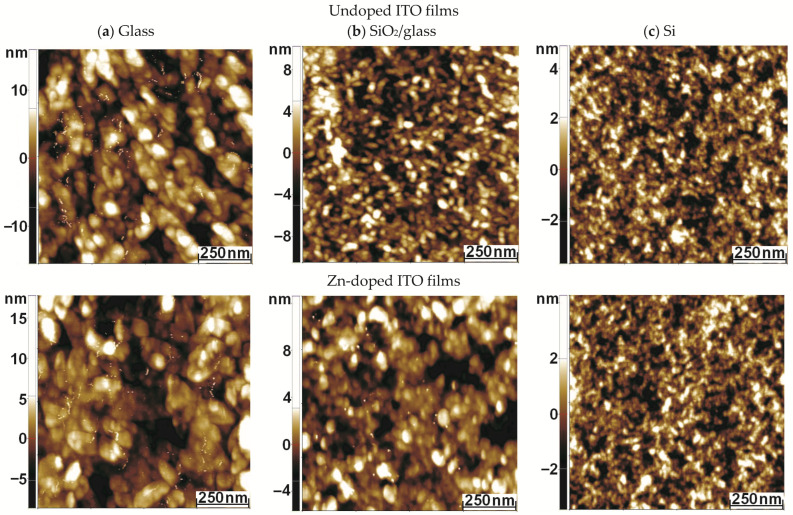
Topographic 2D AFM images at the scale of 1 µm × 1 µm, for undoped (first row) and ITO:Zn films (second row) deposited on three different substrates: (**a**) glass; (**b**) SiO_2_/glass; (**c**) Si.

SEM investigations highlighted continuous and homogeneous coatings for both the undoped and the ITO:Zn films on the Si substrate. To investigate in more detail the morphology of the ITO-based films, on the three substrates used, AFM measurements were performed at the scale of 1 µm × 1 µm (Figure 6a–c).

**Figure 7:**
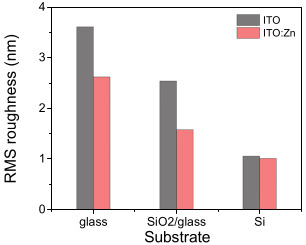
RMS roughness of ITO films before and after Zn doping, as a function of the substrate used: glass, SiO_2_/glass, and Si.

As can be observed, the morphological characteristics depended on the presence of the dopant and on the substrate type used. Both undoped and Zn-doped ITO films deposited on glass exhibited a similar texture, with the superficial grains gathered in small clusters, which alternated with random voids. Much smaller particles were observed on the surface of undoped ITO film on SiO_2_/glass substrate in comparison with ITO:Zn film deposited on the same substrate. Some differences could also be noted for the films deposited on the Si substrate, where smaller grains in the series were noted for both films (doped and undoped). The root-mean-square (RMS) roughness (Figure 7), assessed from the AFM images, exhibited low values and decreased after doping, relative to the substrate used, in the sequence of glass > SiO_2_/glass > Si. The maximum roughness values did not exceed 4 nm. Such low roughness could also be an advantage from electrical and optical points of view, as surface charge and light scattering, respectively, are limited by corrugation, thus promoting good surface conductivity and high transmittance. This could be particularly attractive for TCO applications.

### 3.6. Optical Characterization

**Figure 8:**
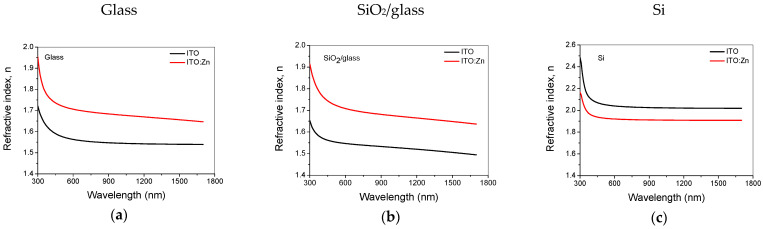

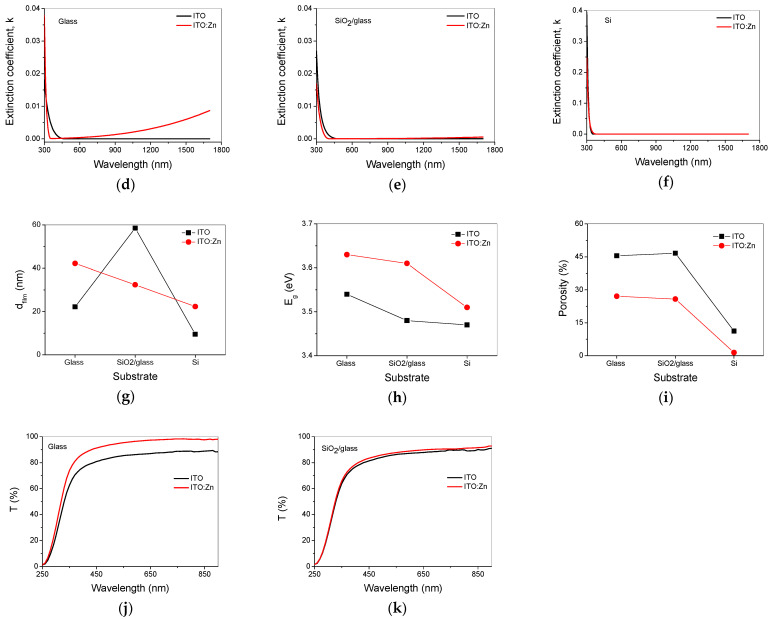
Optical constants—n, k (**a**–**f**), thickness—d (**g**), optical band gap—E_g_ (**h**), porosity—P (**i**), and transmission—T (**j**,**k**) resulting from ellipsometric measurements and analysis of undoped and doped ITO thin films.

The influence of the dopant (Zn) on the optical properties of the ITO thin films was investigated using SE in the UV/Vis/NIR spectral range (300–1700 nm). The layer thicknesses, the optical constants (refractive index—n and extinction coefficient—k), and the optical bandgap (E_g_) were obtained by fitting the experimental data using a three-layer model (surface roughness layer/ITO film/substrate) containing Tauc–Lorentz (to describe the absorption in the UV) and Drude (to describe the effect of the electric charge carriers on the dielectric function when passing from the visible to the infrared wavelength range) oscillators [66]. The effective medium approximation (EMA) was used to model the roughness layer, which was considered a mixture of 50% material (film) and 50% voids (air) [67]. The quality of the fits was evaluated through a regression analysis of the optical data, based on MSE [66]. The refractive index (Figure 8a–c), the absorption coefficient (Figure 8d–f), and the film thickness (Figure 8g) values were obtained from the best fit. The thickness for all samples decreased after thermal treatment.

The optical bandgap energy of the films was calculated using the spectral dependence of the extinction coefficient, k, derived from the absorption coefficient *α* values (α=4πk/λ) by Tauc plots of (*αhν*)^1/2^ versus photon energy (*hν*) for indirect transitions [68] (Figure 8h).

The porosity of the films (Figure 8i) was calculated using the following formula [69]:

(2) $$P=\left[1-\frac{n^{2}-1}{n_{d}^{2}-1}\right]\times 100\;\left(\%\right),$$

where n_d_ = 1.92 is the refractive index of the pore-free ITO (at λ = 500 nm) from the WASE program [66], and n is the refractive index of undoped or Zn doped ITO films at the same wavelength. As can be seen from Figure 8i, the films deposited on glass and SiO_2_/glass were more porous in comparison with those deposited on Si (the film behavior followed the substrate porosity), in agreement with the results obtained from the AFM analysis.

The transmittance of the ITO films was obtained from ellipsometric measurements, and the results are presented in comparison with those obtained after doping of the films, for the two transparent substrates used (glass and SiO_2_/glass). As can be seen in Figure 8j,k, the transmission value reached 90% after doping. This increase in transmission was attributed to the low refractive index, the high porosity, the small crystallite size, and the low surface roughness with good homogeneity of the films as evidenced from the XRD, AFM, and SEM results.

It was observed that Zn-doping of ITO reduced the bandgap of ITO:Zn thin films as compared to ITO thin films and could be attributed to the Burstein–Moss shift in the visible spectral regions.

### 3.7. Raman Spectroscopy

**Figure 9:**
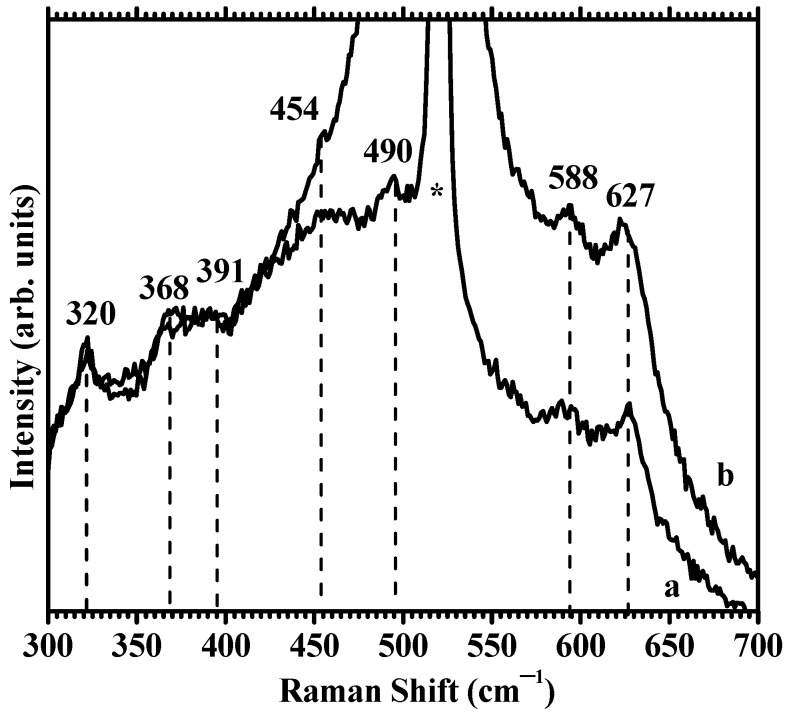
Raman spectra of (**a**) ITO film on Si and (**b**) ITO:Zn film on Si.

The vibrational properties of Zn-doped and undoped ITO films at room temperature were investigated using micro-Raman spectroscopy. ITO and cubic In_2_O_3_ structures belonged to the Ia3, Th7 space group. Factor group analysis predicted up to 22 Raman active modes: 4Ag + 4Eg + 14Tg. Seven modes could be identified in the Raman spectra of the ITO film in the range 300–800 cm^−1^; their positions are marked with lines as a guide to the eye in Figure 9a. The reduced thickness (~30 nm) of the ITO films led to the reduced intensity of the observed ITO bands at ~320 (1), 368 (2), 391 (3), 454 (4), 490 (5), 588 (6), and 627 (7) cm^−1^, compared to the much stronger Si band at 520 cm^−1^.

Raman vibration modes of cubic In_2_O_3_ have been reported at 308, 365, 471, 504, 637, and 707 cm^−1^ [70], at 307, 368, 497, and 632 cm^−1^ [71], at 307 366, 497, and 630 cm^−1^ [72], at 307, 366, 407, 495, 560, and 630 cm^−1^ [73], and at 307, 366, 495, 517, and 631 cm^−1^ [74]. Reported modes correspond with the intense modes 1–7 and the weak modes 2–3–5 observed here, with slightly displaced positions compared to cubic In_2_O_3_. For tin-doped ITO, additional vibration modes at 433–451 and 584 cm^−1^ (modes 4–6 observed here) have been reported [58,74].

Figure 9b shows the Raman spectrum of ITO:Zn film on Si. The spectrum contains the same features observed in the ITO film, except for the presence of a strong broad band, centered at 505 cm^−1^, overlapping with the Si main band, as well as with ITO mode 5. This additional feature was attributed to Zn–O bonds.

In UV-excited (λ_exc_ = 325 nm) Raman scattering of ZnO with wurtzite structure, the exciting photon energy is resonant with the electronic interband transition energy of ZnO, making the first-order polar A1 (LO) phonon the dominant mode [75]. The position of the observed wide band at 505 cm^−1^ in ITO:Zn was significantly red-shifted compared to the allowed A1 (LO) band position at 575 cm^−1^ in ZnO [76,77]. Zn^2+^ in wurtzite adopts a tetrahedral coordination with four oxygen atoms and a Zn–O bond distance in the theoretical structure of 1.95 Å. Reported values range in the interval 1.95–2.01 Å [78]. The average In–O bond distance in the indium oxide theoretical structure is 2.18 Å [79]. Therefore, the broad and red-shifted Zn-O mode observed in Zn:ITO can be explained by an increased Zn–O bond length in Zn-substituted tetrahedral sites of ITO lattice, as Zn^2+^ are forced to occupy sites in the ITO lattice with longer Zn–O bond length than in ZnO. This explanation is in agreement with the small decrease in the interplanar spacing caused by Zn incorporation in the Zn:ITO lattice observed by XRD.

### 3.8. Electrical Characterization (HE Measurements)

The electrical results carried out on ITO and ITO:Zn sol–gel films deposited on all three substrates were obtained through HE measurements in Van der Pauw configuration. The obtained Hall parameters (resistivity (ρ), mobility (µ), carrier concentration (N), and conductivity (σ) were compared with those obtained by modeling SE data using the Drude model [66,80], and the results are presented in Table 3.

It is known that there are two main sources of free electrons in ITO films, which act as donors: (a) oxygen vacancies (native defects), and (b) Sn^4+^ on a substitutional In^3+^ site (extrinsic defects). For the zinc-doped samples, a slight decrease in resistivity could be observed. There was also an increase in resistivity depending on the substrate used, following the sequence of glass > SiO_2_/glass > Si, which could be related to the film thickness (see Figure 8g). As also observed by [80], the results obtained from SE and HE measurements were not quite similar, although they remained in the same order of magnitude.

However, it may be taken into account that SE data modeling is difficult at low charge carrier concentrations (N < 10^20^ cm^−3^) [80]. This issue could be overcome by obtaining a thicker film. Overall, the results obtained through HE measurements and SE data analysis were in good agreement; some differences may have stemmed from the mathematical modeling of SE data with several oscillators (e.g., Tauc-Lorentz, Drude).

Table 4 shows the optical and electrical properties of our undoped and Zn-doped ITO thin films in comparison with those reported in the literature.

Regarding optical performance, all ITO thin films (doped and undoped) showed high values of transmittance, with the highest values being obtained for the Zn-doped ITO thin films [49,57,81,82]. These differences appeared as a result of the different synthesis and annealing conditions, which led to varying thickness and microstructure (in terms of porosity and morphology). The porosity of nanostructures (powders or films) prepared using the sol–gel method is well known [83], and papers on the matter have been published since many years ago [84,85]. In the case of thin films obtained using the sol–gel method, the low porosity could have been induced by successive thin-layer deposition followed by the corresponding thermal treatment [86,87]. As shown in our previous studies [59,61], the concentration of the precursor solution, the nature/porosity of the substrate, and the number of the deposited layers have an important effect on the crystallinity and the crystallite size [84]. Accordingly, the sol concentration and the viscosity of solution can influence the thickness and porosity of the films. It was also observed that a lower concentration of the solution could improve the wetting of the substrate, which is of a great importance to the multilayer deposition [61]. In order to obtain polycrystalline films with lower porosity, a consolidation treatment (260 °C) of the prepared films was performed before the final annealing (400 °C). The different values of the film’s porosity may be related to the reactivity of the substrate. However, the porosity values provided in Figure 8i strongly depend on the refractive index value (at λ = 500 nm) of the pore-free ITO film, as indicated by Equation (2). Therefore, we can consider the trend of the obtained values in the sense that the porosity of the films deposited on glass and SiO_2_/glass was higher than the porosity of the films deposited on Si, and the porosity of the doped films was even lower.

The electrical properties of the thin films can be improved by decreasing the electrical resistivity. According to the literature, post-annealing treatment in a reducing gas (Ar plasma treatment) is an important step to obtain low values of the resistivity without morphological changes [88,89]. Thus, in terms of resistivity, the experimental results of this work are lower compared to those reported in other studies. The electrical parameters such as carrier concentration and conductivity were enhanced by Zn doping, in good agreement with the most recent work regarding sol–gel Zn-doped ITO thin films [57].

Moreover, as compared with the paper mentioned before, we also investigated the influence of the substrate on the electrical properties of Zn-doped ITO, as a result of our previous study regarding pure ITO thin films [59].

### 3.9. Impedance Spectroscopy Measurements

#### 3.9.1. Nyquist Plots and Equivalent Circuit

The impedances $Z=Z^{\prime }+jZ^{\prime \prime }$ (Z′ and Z″ being, respectively, the real and imaginary components) were represented using Nyquist plots (Z″ vs. Z′). The software Z-view was used to fit the impedances of specific electrical circuits to the Nyquist experimental data (Nyquist representations).

**Figure 10:**
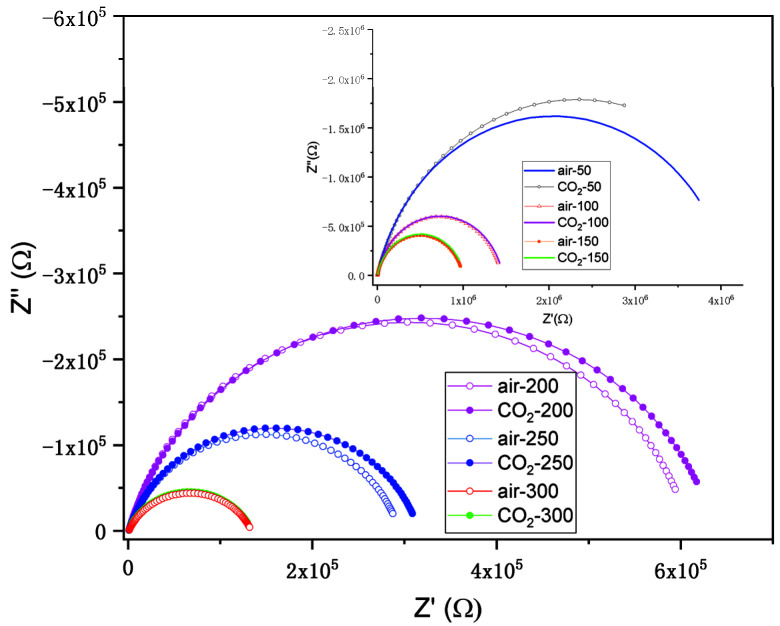
Nyquist representation of the impedance plots of the ITO/Si film in air and in CO_2_ (1000 ppm in air) at different temperatures (100–300 °C).

**Figure 11:**
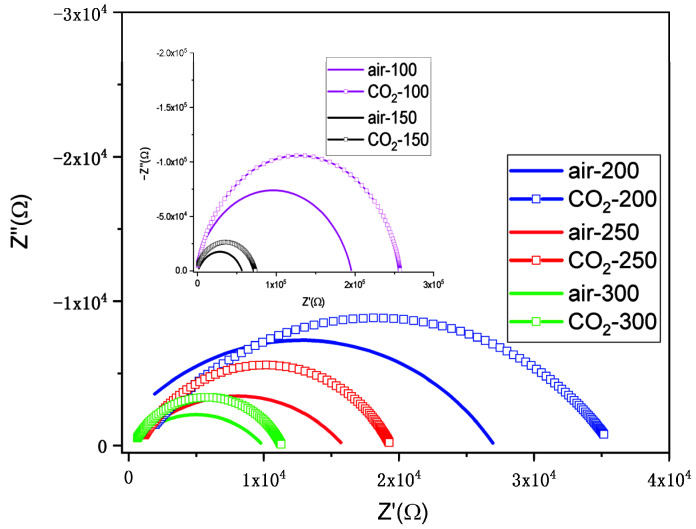
Nyquist representation of the impedance plots of the ITO:Zn/Si film in air and in CO_2_ (1000 ppm in air) at different temperatures (100–300 °C).

In Figure 10 and Figure 11, the impedance plots (Nyquist representation) for the ITO/Si and ITO:Zn/Si films in air and in CO_2_ (1000 ppm in air) at different temperatures (100–300 °C) are presented.

In real cases, the shape of the Nyquist plot does not always show a perfect semicircle as observed for a pure capacitor. Therefore, it is necessary to replace the capacitor (C) with a constant-phase element (CPE) to compensate for the depression of the semicircle of frequency dispersion resulting from the experiment. This can be related to the surface inhomogeneity, surface roughness, electrode porosity, surface disorder, geometric irregularities, etc. The impedance of the CPE is given by the following equation:

(3) $$Z_{\mathrm{CPE}}=\frac{1}{Q{\left(i\omega \right)}^{n}},$$

where ω is the angular frequency, and Q and n are frequency-independent constants, while the constant-phase element is defined by the two values Q and n. When n = 1, the CPE behaves as a pure capacitor, while, when n = 0, the CPE behaves as a pure resistor.

It was observed for the ITO/Si film that the difference between the behavior in air and in CO_2_ was very small.

The equivalent circuit used for fitting the experimental data was a resistor connected to CPE in parallel (R-CPE element). The fitted parameters related to impedance measurements for ITO:Zn/Si film are presented in Table 5.

#### 3.9.2. Activation Energy

Activation energies (Ea) of the conductivity were calculated from the Arrhenius plots according to the following equation:

(4) $$\ln\left(\sigma \right)=\;\mathrm{lnA}\;–\;\frac{E_{a}}{\mathrm{kT}},$$

where σ is the conductivity, T is the absolute temperature, A is the pre-exponential factor, and k is the Boltzmann constant.

**Figure 12:**
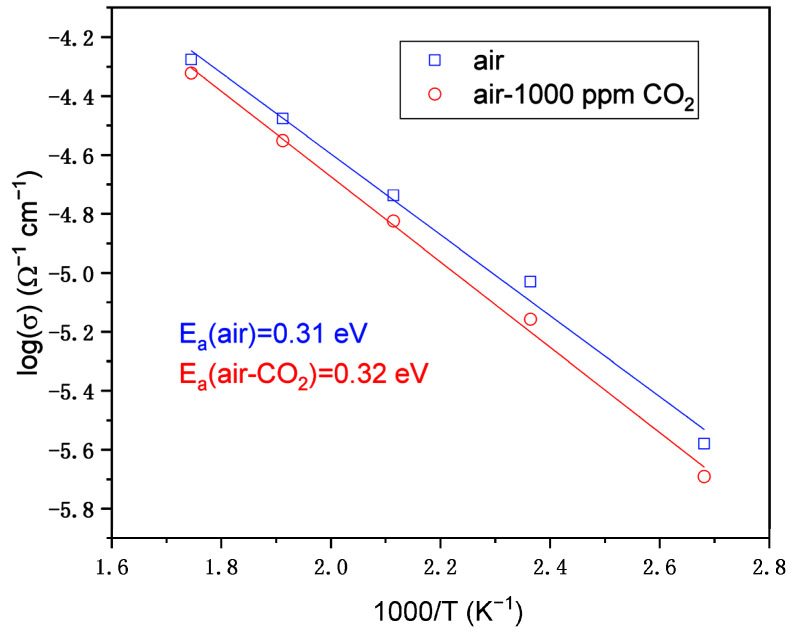
Arrhenius plot of DC conductivity from impedance spectroscopy.

From Table 5, the resistance values can be used to determine the activation energies of the conductivity. The conductivity (σ) vs. 1000/T plot of ITO:Zn/Si film at different temperatures (100–300 °C) is presented in Figure 12. The activation energy value was approximately the same (0.31 vs. 0.32 eV) as the small amount of CO_2_ did not influence the behavior of the film in air.

#### 3.9.3. Electrical Response to the CO_2_ Sensing Gas

CO_2_ is considered a weak reducing gas and produces an increase in resistivity by interacting with surface oxygen species. It was observed that the value of the resistance in the gaseous atmosphere increased upon exposure to CO_2_ (1000 ppm in air) over the entire temperature range (Table 6).

The film showed the highest sensor response of 1.34 at low temperature (150 °C).

## 4. Conclusions

Undoped and Zn-doped ITO films were obtained through five successive depositions using the sol–gel and dipping methods on three different types of substrates: glass, SiO_2_/glass, and Si. 

Morphological investigations using SEM, TEM, and AFM analyses proved that the ITO films were continuous and very thin (~30 nm for both Zn-doped and undoped), with low surface roughness. The RMS roughness, evaluated from AFM images, decreased after doping and with respect to the substrate in the sequence glass > SiO_2_/glass > Si. The weight ratio of Zn/(In + Sn), calculated from EDX (SEM) spectra, was found to be 4% in the doped films, for the samples deposited on Si, where Na screening (from the substrate) was not an issue; this was confirmed by EDX (TEM) profiles showing that Zn distribution along the film was uniform. The Raman study confirmed the formation of ITO films and the incorporation of Zn in the doped ITO:Zn film.

All thin films were polycrystalline, indexed to the cubic bixbyite-type In_2_O_3_ structure. No other diffraction lines (no secondary phases) were present, indicating that the Zn dopant was completely incorporated into the Sn-doped In_2_O_3_ lattice. An increase in crystallinity for the Zn^2+^-doped ITO thin films could be noted.

SE evidenced an increase in the optical bandgap by doping and a clear dependence on the type of substrate used; the films deposited on glass and SiO_2_/glass are more porous in comparison with those deposited on Si, with a transmission value above 90% after doping.

Regarding the electrical properties, there was an increase in resistivity depending on the substrate used, following the sequence of glass> SiO_2_/glass > Si, which could have been related to the film microstructure (thicknesses, porosity, and roughness).

The multifunctional characteristic of undoped and Zn-doped ITO films was examined with respect to TCO characteristics (optical properties—transmission and optical bandgap, and conductivity), as discussed above, as well as gas-sensing experiments toward CO_2_ detection (1000 ppm in air). Gas-sensing experiments were accomplished using the four-point probe method on a Probostat standard cell via impedance spectroscopy, in the temperature range of 100–300 °C. The activation energy was found to be the same (0.31/0.32 eV) as the small amount of CO_2_ did not influence the behavior of the film in air, but the value of resistance in the gaseous atmosphere increased upon exposure in CO_2_ (1000 ppm in air) across the whole temperature range. The best response in terms of maximum sensitivity was obtained at 150 °C for the doped sample.

## Figures and Tables

**Table 1:** Cationic elemental composition (Sn, In, and Zn) (at.%) of the films deposited on Si.

	In (%)	Sn (%)	Zn (%)
ITO	84	16	0
ITO:Zn	81	15	4

**Table 2:** Structural parameters and crystallite size.

Sample Name	2θ (°)	d (Å)	FWHM (°)	D (Å)
SiO_2_/Glass				
ITO	30.68 (5)	2.912 (4)	0.77 (5)	112 (7)
ITO:Zn	30.84 (2)	2.897 (2)	0.94 (2)	91 (2)
Si (100)				
ITO	30.70 (5)	2.910 (4)	0.82 (5)	104 (6)
ITO:Zn	30.82 (3)	2.899 (2)	0.94 (2)	92 (2)
Glass				
ITO	30.73 (7)	2.907 (6)	0.90 (7)	95 (7)
ITO:Zn	30.83 (3)	2.898 (3)	0.95 (3)	91 (3)

**Table 3:** Comparison of the electrical parameters obtained through SE data modeling and HE measurements.

Sample	Substrate	ρ (×10^−2^ Ω·cm)		µ (cm^2^/V·s)		N (×10^19^ cm^−3^)		σ (1/Ω·cm)	
		HE	SE	HE	SE	HE	SE	HE	SE
ITO:Zn	Glass	1.32	1.46	9.49	14.63	3.53	2.91	75.75	68.49
	SiO_2_/glass	1.77	5.42	16.67	9.06	3.9	1.26	56.49	18.45
	Si	2.31	2.56	9.50	10.56	3.83	2.30	43.29	39.06
ITO	Glass	2.83	2.31	11.2	7.26	1.18	3.18	35.33	43.29
	SiO_2_/glass	2.91	2.51	15.6	8.72	5.38	3.02	34.36	39.84
	Si	4.36	2.17	12.2	9.55	7.17	3.01	22.93	46.08

**Table 4:** Comparison of pure and Zn-doped ITO thin films properties.

Sample	Substrate	Average Transmittance (%)	Resistivity (10^−2^ Ω·cm)	Mobility (cm^2^/V·s)	Carrier Concentration (10^19^ cm^−3^)	Ref.
ITO	Glass	>85	70	7.6	0.61	[81]
ITO	Glass	74	9.24	*	*	[49]
ITO	Glass	>85	0.41	14.8	10.2	[82]
ITO	Glass	77	*	1.99	3.6	[57]
Zn-doped ITO	Glass	>80	*	1.01	4.3	[57]
ITO	Glass	80	2.83	11.2	3.01	This work
Zn-doped ITO	Glass	90	1.32	9.49	2.91	

* Not mentioned.

**Table 5:** The fitted parameters with the equivalent circuit for ITO:Zn/Si film.

Gaseous Atmosphere/Temperature	Rp	CPE-T	CPE-P
Air/100 °C	199,180	9.02 × 10^−11^	0.81381
Air/150 °C	56,072	1.83 × 10^−9^	0.7087
Air/200 °C	28,605	5.58 × 10^−9^	0.60087
Air/250 °C	15,693	3.97 × 10^−8^	0.52506
Air/300 °C	9886	9.50 × 10^−8^	0.5201
Air–1000 ppm CO_2_/100 °C	257,480	5.50 × 10^−11^	0.87624
Air–1000 ppm CO_2_/150 °C	75,274	2.12 × 10^−10^	0.77648
Air–1000 ppm CO_2_/200 °C	34,974	1.77 × 10^−8^	0.59574
Air–1000 ppm CO_2_/250 °C	18,648	8.24 × 10^−9^	0.68611
Air–1000 ppm CO_2_/300 °C	10,988	9.64 × 10^−9^	0.69566

**Table 6:** ITO:Zn/Si film sensor response at 1000 ppm CO_2_ for different operating temperatures.

Temperature (°C)	Response (R_CO2_/R_air_)
100	1.29
150	1.34
200	1.22
250	1.19
300	1.11

## Data Availability

The data presented in this study are available on request from the corresponding author.
